# Defining Sarcopenia Using the Third Lumbar Vertebra (L3) Skeletal Muscle Index (L3SMI): Establishing Imaging Biomarker Standards for the Middle Eastern Population

**DOI:** 10.7759/cureus.103200

**Published:** 2026-02-08

**Authors:** Abdulmalek Alzahrani, Badr Bannan, Mohammad Alsayed, Zergham Zia

**Affiliations:** 1 Department of Radiology, King Faisal Specialist Hospital and Research Centre, Jeddah, SAU

**Keywords:** diagnostic criteria, imaging biomarkers, l3 skeletal muscle index, middle eastern population, sarcopenia

## Abstract

This study aimed to define sarcopenia criteria using the third lumbar vertebra (L3) skeletal muscle index (L3SMI) in Middle Eastern individuals. In a retrospective review, abdominal CT imaging from 200 kidney donors was analyzed. Demographic data, BMI, body surface area, L3 skeletal muscle area (L3SMA), and L3SMI were evaluated. Mean ± SD L3SMA values were 98.63 ± 14.9 cm² for females (n = 48) and 157.52 ± 25.6 cm² for males (n = 152); corresponding L3SMI values were 40.9 ± 5.6 and 55.3 ± 8.8 cm²/m², respectively. Applying Western sarcopenia cutoffs classified 47.9% of females (n = 23/48) and 26.3% of males (n = 40/152) as sarcopenic. Defining cutoffs as two SDs below the mean yielded 29.7 cm²/m² for females and 37.7 cm²/m² for males. These gender-specific cutoffs may aid in the diagnosis of sarcopenia, support clinical risk stratification, and inform future population-based screening strategies in the Middle Eastern region. Larger multiethnic studies are warranted.

## Introduction

Sarcopenia is a strong prognostic indicator linked to higher morbidity and mortality in patients with cancer [1-6], surgical oncology cases [7-10], liver transplantation [11], patients undergoing emergency surgery [12], intensive care admissions [13], and chronic diseases such as cirrhosis [14]. Defining accurate, population-specific thresholds is essential to guide clinical decision-making, resource allocation, and regional public health strategies for early sarcopenia identification. Most reference values were developed in Western cancer cohorts and may not account for ethnic differences in baseline muscle mass, leading to misclassification when applied to Middle Eastern individuals [6-8].

CT-based estimation of sarcopenia using the third lumbar vertebra (L3) skeletal muscle index (L3SMI) has gained widespread use since its introduction by Prado et al. [1]. L3SMI is calculated by dividing the skeletal muscle cross-sectional area at mid-L3 (cm²) by height squared (m²) and is expressed in cm²/m². Reference values for sarcopenia are defined as <38.5 cm²/m² for females and <52.4 cm²/m² for males. However, these values were predominantly derived from Western populations of patients with cancer and have not been consistently applied across different ethnic groups [3,15,16].

Globally, sarcopenia affects 10-20% of older adults, and its prevalence increases with age. In the Middle East, limited data exist, but rising obesity rates and physical inactivity may contribute to higher unrecognized prevalence, underscoring the need for regional diagnostic criteria [17,18-20].

This study retrospectively analyzes a cohort of healthy individuals undergoing living donor nephrectomy as part of their standard preoperative assessment. This cohort provides a unique opportunity to analyze standardized abdominal CT imaging in a healthy population. Determining L3SMI in this group will reveal its distribution in a healthy population. Gender-specific thresholds, defined as two SDs below the mean, can establish sarcopenia reference values for this population. Conducted at a large live donor renal transplant center in the Middle East, this study defines sarcopenia in the local population.

## Materials and methods

Patient population

This study was Institutional Review Board-approved (approval 2023-123). Two hundred consecutive kidney donors undergoing preoperative imaging from January 2016 to December 2017 were retrospectively reviewed. Donors were included if they were ≥18 years of age, had completed the standard living donor workup, and were deemed medically fit for donation. Exclusion criteria included known kidney disease, diabetes mellitus, uncontrolled hypertension, clinically significant cardiovascular disease, active or recent malignancy, and any other condition precluding donation according to institutional transplant protocols.

CT morphometric analysis

**Figure 1:**
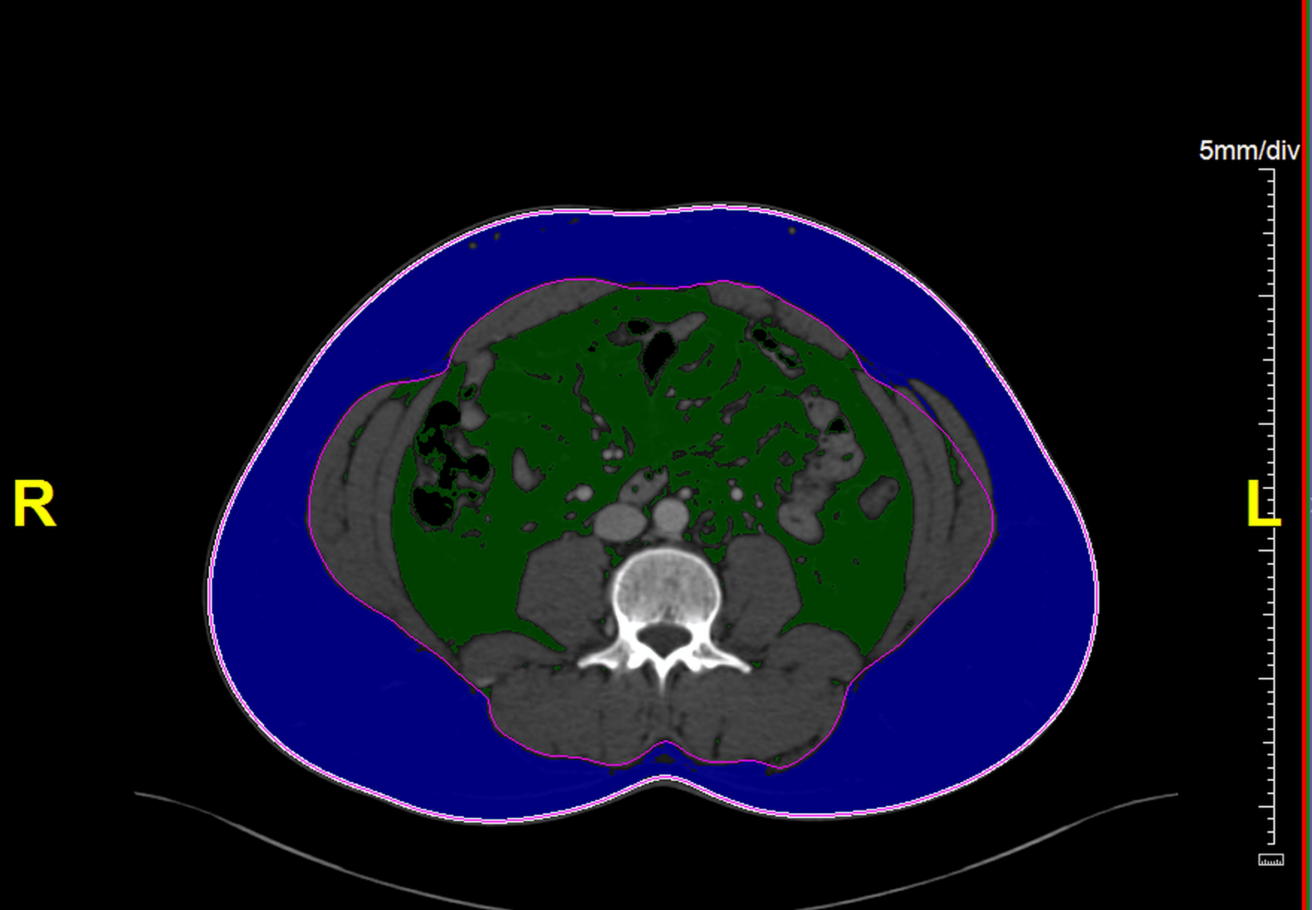
CT scan at the mid-L3 level showing subcutaneous fat (blue) and visceral fat (green) areas In this example, visceral fat measures 140 cm², and subcutaneous fat measures 286 cm². Adipose tissue was identified using CT HU values of -190 to -30 HU. L3, third lumbar vertebra

Electronically archived CT images were analyzed using the TeraRecon Aquarius workstation (TeraRecon Inc., San Mateo, CA, USA). A transverse CT image was selected from the mid-L3 level using multiplanar reformats. The automated fat volume tool was used to calculate subcutaneous and visceral fat content (Figure 1).

**Figure 2:**
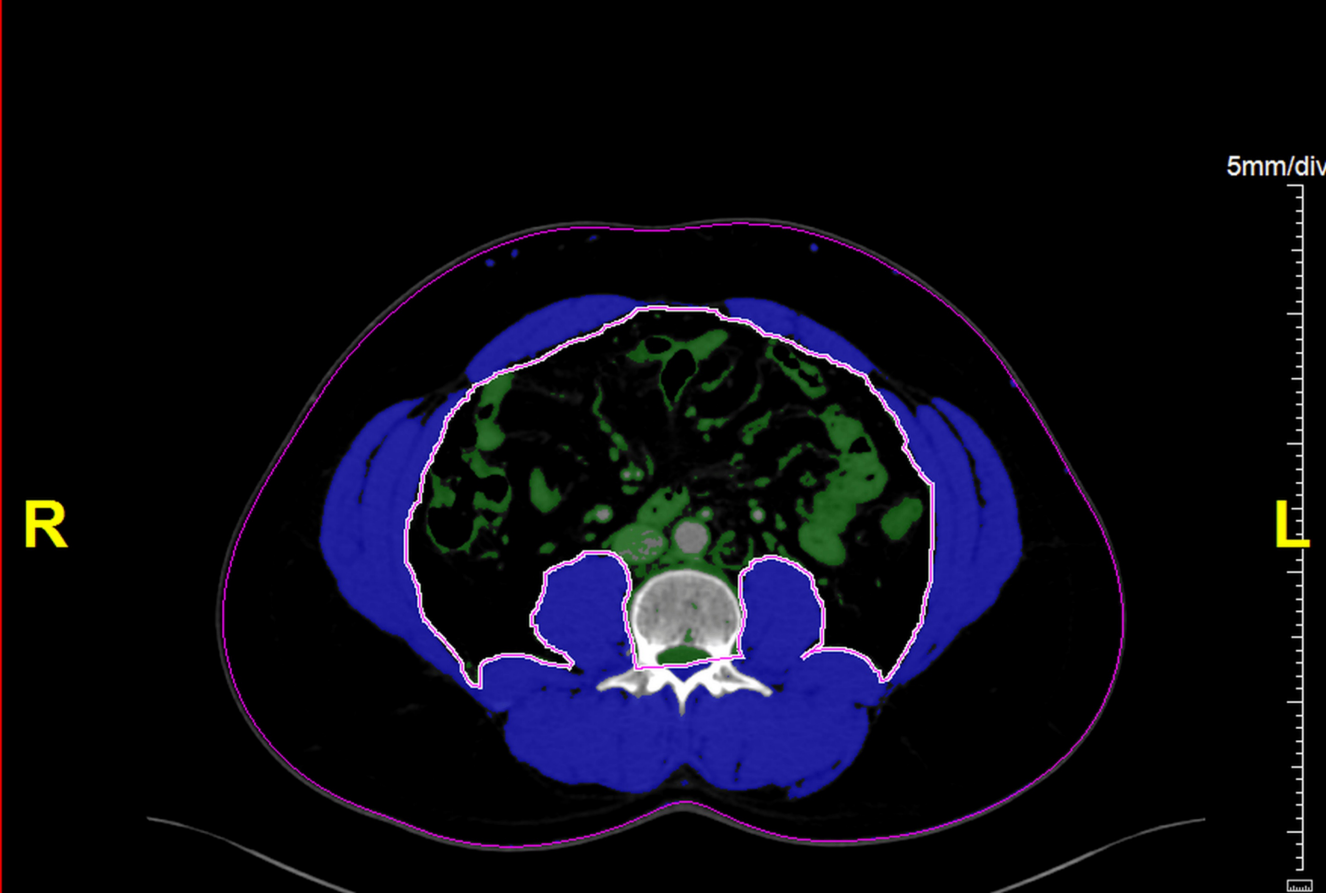
CT scan at the mid-L3 level for L3SMA assessment Skeletal muscle is highlighted in blue, with HU values set at -29 to +150 HU. In this example, L3SMA measures 155 cm². L3SMA, third lumbar vertebra skeletal muscle area

The region of interest was adjusted to include the skeletal muscles (paraspinal, psoas, transversus abdominis, internal and external obliques, and rectus abdominis). Skeletal muscle was identified using HU values of -29 to +150 HU (Figure 2).

Statistical methods

Demographic and clinical characteristics were described for the entire sample and separately for females and males. Means and SDs were reported for normally distributed variables. Normality was assessed using the Shapiro-Wilk test. Comparisons of means and medians, graphical visualizations, and assessments of skewness and kurtosis were also employed to evaluate the distribution of continuous variables.

The distributions of skeletal muscle area (SMA) and skeletal muscle index (SMI) were described using percentiles (P5, P10, P25, P50, P75, P90, and P95). Pearson correlation coefficients were used to assess relationships between age and BMI for females and males. Outliers were identified using box-and-whisker plots. Scatterplots and linear regression were used to examine correlations of age and BMI with skeletal muscle parameters. Regression assumptions were verified through scatterplots of residuals versus predicted and independent values, Durbin-Watson statistics, normal probability plots, and histograms of residuals.

All statistical analyses were performed using SAS version 9.4 (SAS Institute Inc., Cary, NC, USA), with statistical significance set at α = 0.05.

## Results

The study included 200 healthy kidney donors, 75% of whom were male (n = 150). The mean age ± SD of the study population was 31.1 ± 7.9 years, with a range of 17.0-53.0 years (Table 1). The overall mean BMI was 27.0 ± 4.9 kg/m². Age, BMI, height, and weight by gender are also shown in Table 1. Gender-specific sarcopenia parameters across age quartiles are summarized in Table 2.

**Table 1:** Study population characteristics Data are presented as mean ± SD. Statistical significance was set at p < 0.05.

Parameter	All participants (n = 200)	Males (n = 150)	Females (n = 50)
Age (years)	31.1 ± 7.9	30.9 ± 7.6	31.7 ± 8.8
Height (m)	1.65 ± 0.10	1.69 ± 0.10	1.55 ± 0.10
Weight (kg)	74.2 ± 15.4	76.7 ± 15.8	66.7 ± 11.7
BMI (kg/m²)	27.0 ± 4.9	26.8 ± 4.9	27.7 ± 4.8

**Table 2:** Gender-specific sarcopenia parameters across age quartiles Data are presented as mean ± SD. SMI, skeletal muscle index; SMA, skeletal muscle area

Gender	Age quartile	N	Age mean (years)	Age SD	BMI mean (kg/m²)	BMI SD	SMI mean (cm²/m²)	SMI SD	SMA mean (cm²)	SMA SD
Female	Q1 (≤26)	14	23.07	1.98	27.39	5.3	40.34	5.96	97.43	17.71
Female	Q2 (26-31)	6	28.86	1.86	30.05	5.91	37.38	5.5	98.26	9.99
Female	Q3 (31-35)	14	33.63	1.26	30.34	4.85	40.25	5.03	97.77	13.1
Female	Q4 (>35)	16	40.76	5.12	27.53	3.1	40.58	5.31	106.46	13.51
Male	Q1 (≤26)	36	21.16	2.63	26.07	4.65	55.8	8.05	154.01	28.13
Male	Q2 (26-31)	44	28.33	1.52	27.51	4.38	53.95	8.97	160.43	25.89
Male	Q3 (31-35)	36	32.97	1.06	27.41	4.43	54.41	9.39	152.73	23.34
Male	Q4 (>35)	34	39.87	3.55	27.28	4.85	52.67	9.34	165.33	26.53

**Figure 3:**
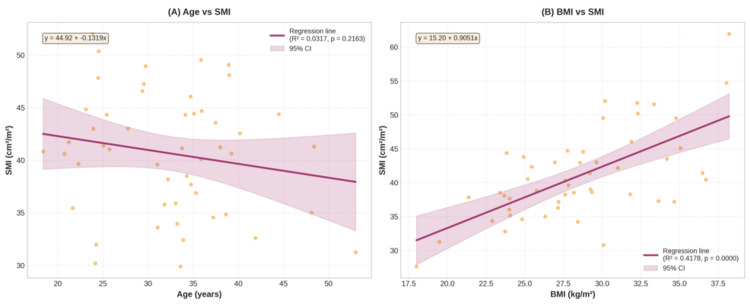
Associations between (A) age and SMI and (B) BMI and SMI in females (n = 50) SMI, skeletal muscle index

**Figure 4:**
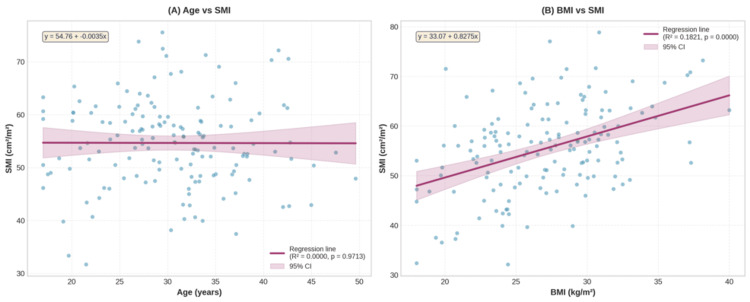
Associations between (A) age and SMI and (B) BMI and SMI in males (n = 150) SMI, skeletal muscle index

The mean L3SMI was 40.9 ± 5.6 cm²/m² for females and 55.3 ± 8.8 cm²/m² for males (Table 3). The P5 for L3SMI was 32.2 cm²/m² for females and 43.0 cm²/m² for males (Table 2). Age was not significantly associated with L3SMI in females (p = 0.8735) or males (p = 0.2437). However, L3SMI increased significantly with BMI in females (R² = 0.2138, p = 0.0007) and males (R² = 0.1484, p < 0.0001) (Figure 3A, 3B, Figure 4A, 4B). 

**Table 3:** Gender-specific percentiles for skeletal muscle parameters, including SMI and SMA Data are presented as mean ± SD. Statistical significance was set at p < 0.05. SMA, skeletal muscle area; SMI, skeletal muscle index

Parameter	Males	Females
Mean SMI (cm²/m²) ± SD	55.3 ± 8.8	40.9 ± 5.6
Mean SMA (cm²) ± SD	157.5 ± 25.7	98.4 ± 14.9
P5 SMI	43	32.2
P5 SMA	119	79.3
P10 SMI	44.7	34.1
P10 SMA	129	81.5
P25 SMI	49.8	37.6
P25 SMA	140	88.4
P50 SMI	54.1	40.6
P50 SMA	157.5	95.3
P75 SMI	60.9	44.1
P75 SMA	175	106
P90 SMI	66.1	47.3
P90 SMA	185.5	121
P95 SMI	70.9	52.2
P95 SMA	192	132

**Figure 5:**
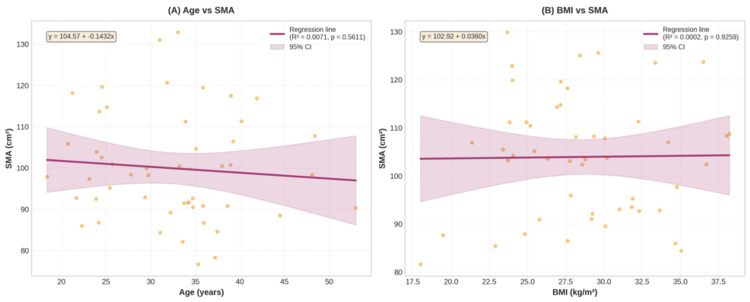
Associations between (A) age and SMA and (B) BMI and SMA in females (n = 50) SMA, skeletal muscle area

**Figure 6:**
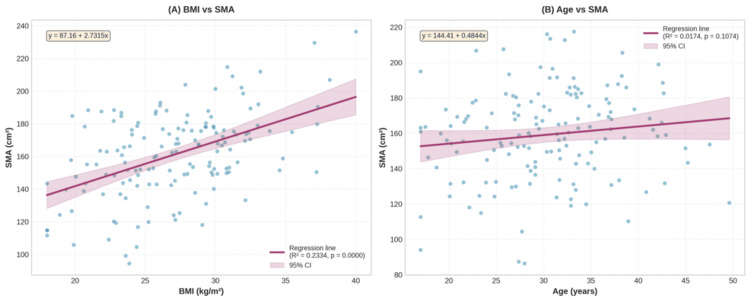
Associations between (A) age and SMA and (B) BMI and SMA in males (n = 150) SMA, skeletal muscle area

The mean L3 SMA (L3SMA) was 98.4 ± 14.9 cm² in females and 157.5 ± 25.7 cm² in males (Table 3). The P5 for L3SMA was 79.3 cm² for females and 119.0 cm² for males (Table 2). Similar to L3SMI, age was not significantly associated with L3SMA in females (p = 0.4394) or males (p = 0.5218). However, L3SMA increased significantly with BMI in females (R² = 0.1045, p = 0.0220) and males (R² = 0.1682, p < 0.0001) (Figure 5A, 5B, Figure 6A, 6B).

## Discussion

Sarcopenia is a progressive skeletal muscle disorder associated with an increased risk of adverse outcomes, including falls, fractures, physical disability, and mortality. Various techniques are used to define sarcopenia, including clinical questionnaires, anthropometry, bioimpedance analysis (BIA), dual-energy X-ray absorptiometry (DEXA), and imaging techniques such as CT, MRI, and ultrasound. The European Working Group on Sarcopenia 2 (EWGSOP2) defines sarcopenia and recommends using the SARC-F clinical questionnaire for screening, followed by clinical muscle strength tests. Confirmation of sarcopenia is achieved through muscle quantification via DEXA or BIA in clinical settings and via DEXA, CT, or MRI in research settings [17]. CT is a widely used clinical tool that provides high-quality quantitative information on muscle composition and distribution, allowing precise measurements of fat and muscle content. CT imaging at the L3 strongly correlates with whole-body muscle mass [18]. The L3SMI, derived from CT, has demonstrated prognostic value in oncology, critical care, and surgical populations [10,15,19]. EWGSOP2 has endorsed CT assessment for sarcopenia research and anticipates its adoption in clinical practice, although no specific L3SMI cutoff values have been universally established.

Defining accurate, population-specific thresholds is essential to guide clinical decision-making, resource allocation, and regional public health strategies for early sarcopenia identification. The L3SMI diagnostic criteria for sarcopenia were introduced in 2008 (females: SMI <38.5 cm²/m²; males: SMI <52.4 cm²/m²) [1], based on survival rates in obese European patients with cancer. These thresholds are widely used in studies to assess outcomes in various clinical settings; however, they may not account for racial and ethnic differences in muscle mass. For instance, Asian populations exhibit up to 17% lower muscle mass compared with Western populations [20], likely affecting L3SMI cutoff values. Studies in other populations have suggested lower cutoffs. In Chinese patients with gastric cancer (n = 937), optimal cutoff values were <34.9 and <40.8 cm²/m² for females and males, respectively [21]. Japanese patients with liver cirrhosis exhibited a sarcopenia prevalence of 11.1%, with mortality-based cutoffs of 29.6 cm²/m² for females and 36.2 cm²/m² for males [22]. In healthy Indian individuals (n = 275), cutoffs defined as two SDs below the mean were <30.2 cm²/m² for females and <36.5 cm²/m² for males [16].

The present study evaluated L3SMI in 200 renal donors at a Saudi tertiary renal transplant center. Renal donors are considered healthy due to extensive predonation screening. Using Western cutoff values, 47.9% of females and 26.3% of males in this healthy cohort would be classified as sarcopenic. However, gender-specific L3SMI cutoffs for the Middle Eastern population, derived from two SDs below the mean in this cohort, suggest that values below 29.7 cm²/m² for females and below 37.7 cm²/m² for males indicate sarcopenia. This study found no significant relationship between age and L3SMI, likely owing to the relatively narrow age range of the cohort.

Limitations of this study include data collection from a single center and a relatively young population. Larger studies involving more diverse age groups and broader populations are needed to validate these cutoff values. Further research should assess their predictive value for clinical outcomes to enhance their utility.

## Conclusions

L3SMI-based thresholds of 29.7 cm²/m² for females and 37.7 cm²/m² for males may define sarcopenia in the Middle Eastern population. These gender-specific cutoffs can aid in the diagnosis of sarcopenia, support clinical risk stratification, and inform future population-based screening strategies in the region. Further multiethnic, age-diverse studies are needed to confirm and refine these values, enhancing their clinical utility.
